# *Theonella*: A Treasure Trove of Structurally Unique and Biologically Active Sterols

**DOI:** 10.3390/md21050291

**Published:** 2023-05-08

**Authors:** Carmen Festa, Simona De Marino, Angela Zampella, Stefano Fiorucci

**Affiliations:** 1 Department of Pharmacy, University of Naples, Via Domenico Montesano, 49, 80131 Naples, Italy; azampell@unina.it; 2 Department of Medicine and Surgery, University of Perugia, Piazza L. Severi, 1, 06132 Perugia, Italy; stefano.fiorucci@unipg.it

**Keywords:** marine sponge, *Theonella*, secondary metabolites, sterols, nuclear receptor ligands, cytotoxic activity, structure–activity relationship (SAR)

## Abstract

The marine environment is considered a vast source in the discovery of structurally unique bioactive secondary metabolites. Among marine invertebrates, the sponge *Theonella* spp. represents an arsenal of novel compounds ranging from peptides, alkaloids, terpenes, macrolides, and sterols. In this review, we summarize the recent reports on sterols isolated from this amazing sponge, describing their structural features and peculiar biological activities. We also discuss the total syntheses of solomonsterols A and B and the medicinal chemistry modifications on theonellasterol and conicasterol, focusing on the effect of chemical transformations on the biological activity of this class of metabolites. The promising compounds identified from *Theonella* spp. possess pronounced biological activity on nuclear receptors or cytotoxicity and result in promising candidates for extended preclinical evaluations. The identification of naturally occurring and semisynthetic marine bioactive sterols reaffirms the utility of examining natural product libraries for the discovery of new therapeutical approach to human diseases.

## 1. Introduction

“Who finds a Theonella, finds a treasure”. It is amazing that even today, worldwide, there is a deep interest in the chemistry and biology of the sponge of the genus *Theonella* (Lithistida, Theonellidae). In the early 1980s, Kashman managed to recognize the potential of this sponge as a source of secondary metabolites with unique chemical structures and very interesting pharmacological activities [1]. This review provides an update on the isolation and semisynthesis of sterols from different species of marine sponges of the genus *Theonella* (order Lithistida, class Demospongiae).

Sponges of the genus *Theonella* have fascinated the scientific community as they have proved to be a prolific source of intriguing secondary metabolites [2,3]. To date, through extensive research on this marine invertebrate, more than 100 metabolites have been reported in the literature, including polyketide macrolides [4,5], linear and cyclic peptides [6,7], depsipeptides [8,9], and uncommon steroids [1,10,11,12,13].

The chemical diversity found in several secondary metabolites from *Theonella* spp. has been ascribed in part to the presence of symbiotic microorganisms [14,15], recognized as the “real chemical factories”.

## 2. Sterols from *Theonella* spp.

In this review, we focus our attention on more than 50 sterols identified from *Theonella* spp., including conicasterols [16], theonellasterols [17], swinhosterols [10], swinhoeisterols [13], and solomonsterols [11]. A majority of these unique chemical compounds are characterized by potent and interesting biological activities such as antimicrobial [17], cytotoxic [9], and in some cases by modulating activity towards metabolic nuclear receptors, mainly the pregnane X receptor (PXR) and the farnesoid X receptor (FXR). In particular, the modulation ranges from the selective antagonism on FXR of theonellasterol (**1**) [18] and selective agonism on PXR of the truncated-sulfated derivatives, solomonsterol A (**47**) and B (**48**) [11], to dual modulation on FXR/PXR of other 4-methylene steroids [19]. The translation potential of these natural compounds was also validated by extensive in vivo pharmacological exploration [19,20].

### 2.1. 4-Exo-Methylene Sterols

The main class of sterols isolated from sponges of the genus *Theonella*, and particularly from *Theonella swinhoei* and *Theonella conica*, are 4-exo-methylene sterols, relatively rare metabolites in nature. The biosynthetically unusual 4-exo-methylene group arises from the oxidative demethylation of the 4,4-dimethyl precursor followed by oxidation and dehydration of the primary alcohol affording the 4-exo-double bond [1].

**Figure 1:**
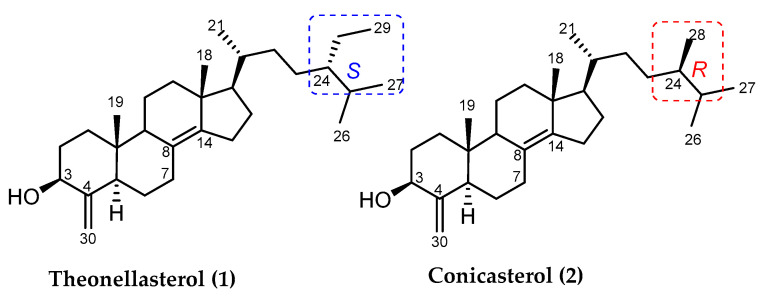
Structural comparison of theonallasterol (**1**) and conicasterol (**2**).

Among 4-exo-methylene sterols, theonellasterol (**1**) and conicasterol (**2**), reported for the first time by Djerassi et al. [1], are also 24-alkylated derivatives, and specifically theonellasterol (**1**) with the (24*S*)-ethyl group represents the biomarker of the species *T*. *swinhoei*, whereas conicasterol (**2**), with the (24*R*)-methyl group, is the biomarker of the *T. conica* species. As structural features, both molecules share the same tetracyclic core bearing the β-hydroxyl group at C-3, the unusual exo-methylene functionality at C-4, and the rare ∆^8,14^ double bond (Figure 1).

**Figure 2:**
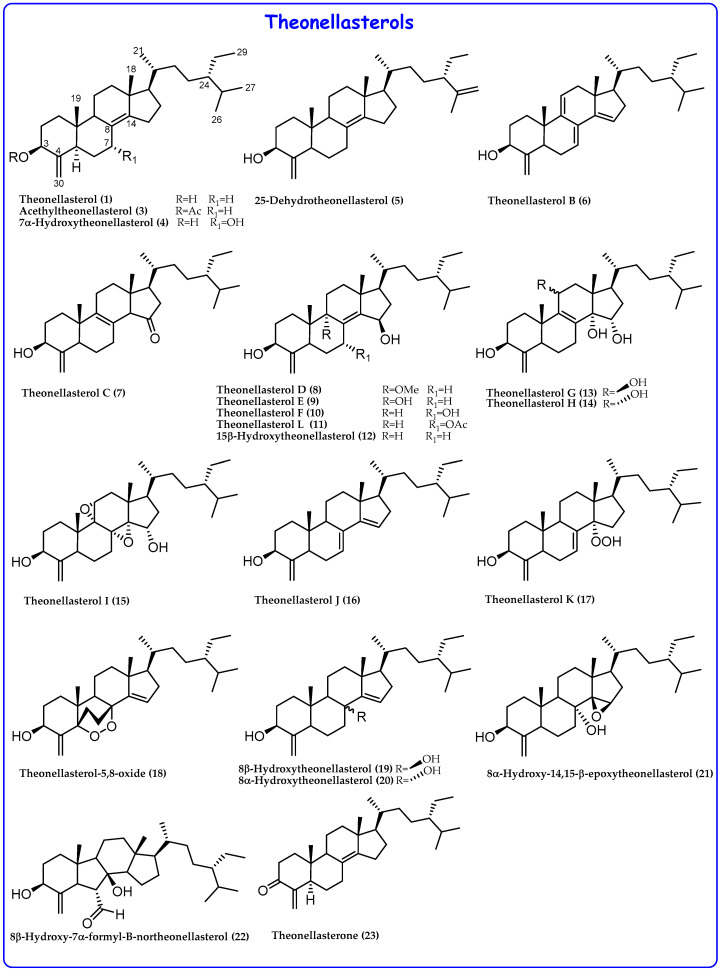
Theonellasterols from *Theonella* spp.

**Figure 3:**
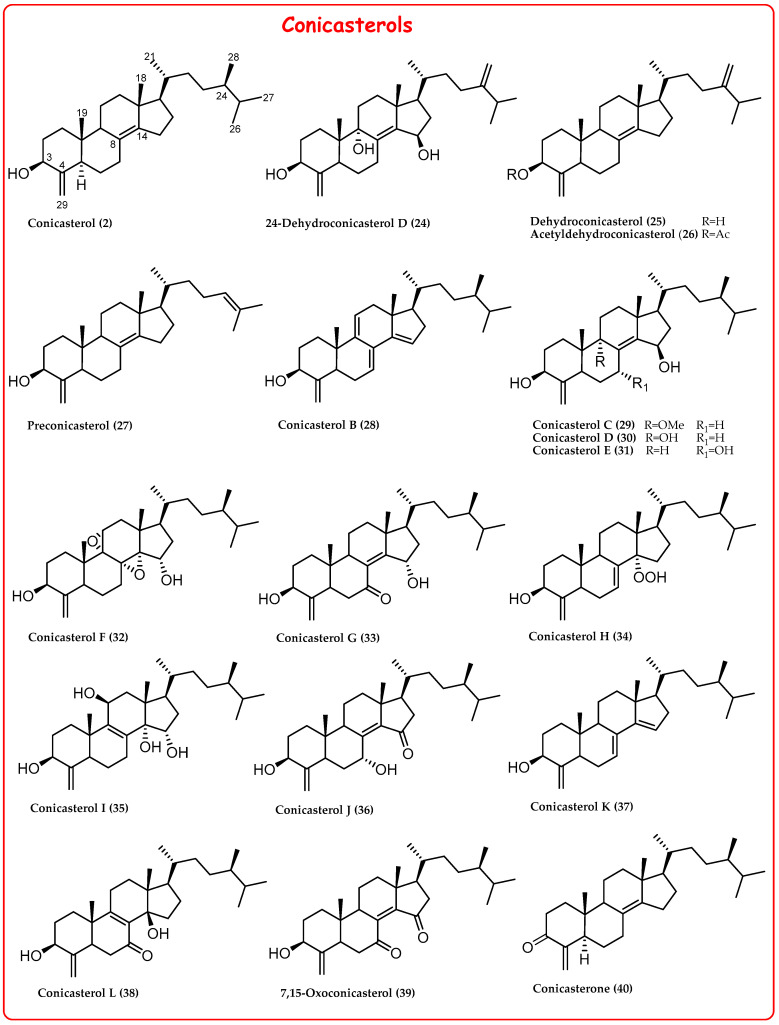
Conicasterols isolated from sponges of the genus *Theonella*.

Different specimens of *Theonella*, collected in different geographic areas, allowed the isolation of a large library of 4-methylene sterols (Figure 2 and Figure 3) featuring more complex functionalizations in the steroidal nucleus, such as:

✓ A keto group at C-3 in theonellasterone (**23**) and conicasterone (**40**);
✓ The presence of additional hydroxyl groups at C-7, C-8, C-9, C-14, or C-15;
✓ The presence of oxygenated functions at C-7 or C-15;
✓ Epoxide groups at positions 8,14, 14,15, or 9,11;
✓ Additional rare double bonds Δ^7,8^, Δ^8,9^, Δ^9,11^, and Δ^14,15^;
✓ A peroxide group at C-14 in theonellasterol K (**17**) and conicasterol H (**34**) and an endoperoxide ring in theonellasterol-5,8-oxide (**18**).

The modifications in the side chains are less common and mainly regard the presence of additional double bonds.

**Figure 4:**
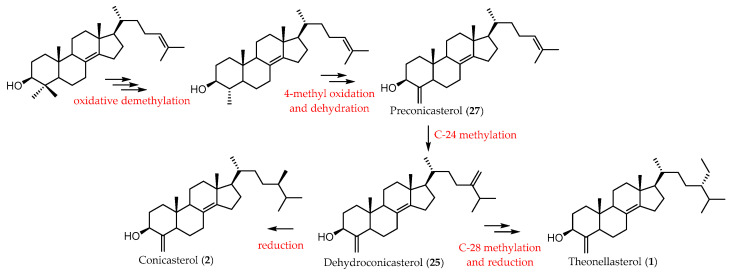
Plausible biogenesis of theonellasterol (**1**) and conicasterol (**2**).

Preconicasterol (**27**), featuring a Δ^24^ double bond, represents the first example of 4-methylene steroid without any branching in the side chain [21] while dehydroconicasterol (**25**) shows an additional exo-methylene group at C-24 [22]. As reported in Figure 4, preconicasterol (**27**) should be considered the biogenetic precursor of the branched dehydroconicasterol (**25**) that, in turn, subjected to reduction and/or transmethylation with *S*-adenosylmethionine, led to the formation of conicasterol (**2**) or theonellasterol (**1**) [21].

From the apolar extracts of *Theonella swinhoei* and *Theonella conica* specimens collected off Kakeroma Island, in 2014, Sepe et al. isolated two 4-methylene steroids modified in the side chain: 24-dehydroconicasterol D (**24**), featuring an additional double bond between C-24 and C-28, and 25-dehydrotheonellasterol (**5**), with the side chain also including an additional C-25 double bond [21].

### 2.2. 4-Exo-Methylene Sterols as Modulators of NRs

Sterols isolated from *Theonella* spp. are shown to possess various pharmacological activities such as anticancer, antimicrobial, and, more recently, modulatory action towards two nuclear receptors, PXR and FXR [11,17,18,19,20,21].

Nuclear receptors (NRs), together with rhodopsin-like GPCRs, are well-recognized molecular targets in drug discovery and are unique to the animal kingdom [23,24]. Indeed, the presence of an ancestral NR has been demonstrated in sponges, the simplest animal organisms, and it is well recognized that there is a close relationship between the complexity of the organism and the diversification of the genes encoding for NR. Moreover, during the evolution along the metazoan tree, both changes in the structural organization of the receptors and their corresponding ligands occurred [25].

NRs are ligand-activated transcription factors that regulate the expression of genes involved in several physiological and physio-pathological processes, including reproduction, metabolism of xeno- and endobiotics, and inflammation [26]. They are characterized by a common organization of several domains, with the most conserved DNA-binding domain (DBD) and ligand-binding one (LBD). LBD accommodates ligands and undergoes conformational changes. NRs are generally found as monomers but function as heterodimer complexes with another nuclear receptor, the retinoid X receptor (RXR), in binding to DNA. In the absence of a ligand, this complex is associated with several corepressors while the binding of a ligand allows the release of the corepressors and the recruitment of coactivators and, consequently, the activation of the transcription machine.

The potentialities of this type of pharmacological targets lie in different factors, such as their ability to respond to specific small molecules, including intracellular metabolites and xenobiotics, their pleiotropic nature that allows a single receptor to influence the expression of many genes, and their involvement in the regulation of several metabolic and inflammatory diseases, including diabetes, dyslipidemia, cirrhosis, and fibrosis.

Among the NRs, the pregnane X receptor (PXR), also known as xenobiotic sensor, is mainly involved in bile acid homeostasis and nowadays is considered as a key factor in bile acid detoxification in liver and in guts. PXR also plays important roles in various pathophysiological processes, such as lipid metabolism, glucose homeostasis, and inflammatory response [27,28], including liver disease and inflammatory bowel diseases (IBD) [29,30]. PXR LBD is larger compared to ones of other NRs and is characterized by hydrophobic residues, allowing the binding of many structurally different ligands, some of them isolated from marine organisms [31].

The farnesoid X receptor (FXR) is a bile acid sensor, regulating bile acid homeostasis and lipid and glucose metabolism. FXR is highly expressed in the liver, intestine, kidneys, and adrenal glands [32,33] and is activated by bile acids, with chenodeoxycholic acid (CDCA, **41**) or 6α-ethyl-chenodeoxycholic acid (6-ECDCA or OCA, **42**) as the most potent endogenous and semisynthetic ligands, respectively. FXR also has an important effect on inflammation. Ligands of this receptor have become promising therapeutic agents for different diseases, such as primary biliary cirrhosis (PBC) and nonalcoholic fatty liver disease (NASH) [34].

Among the 4-methylene steroids, theonellasterol (**1**) represents the first example of a natural highly selective FXR antagonist [18], unlike the most promiscuous guggulsterone [35,36]. Theonellasterol (**1**) has been proven to antagonize FXR transactivation caused by CDCA, reversing the effect of CDCA on the expression of canonical FXR target genes including OSTα, BSEP, SHP, and MRP4. Moreover, theonellasterol (**1**) stabilizes the recruitment of the nuclear corepressor NCoR, thus inhibiting the expression of FXR-regulated genes.

**Figure 5:**
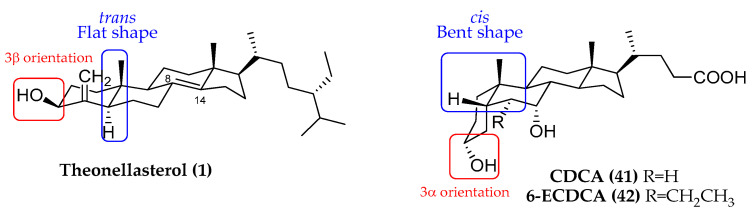
Structural comparison between theonellasterol (**1**) and CDCA.

From a chemical point of view, theonellasterol (**1**) profoundly differs from the endogenous ligand of FXR, CDCA, (Figure 5) mainly in:

✓ The orientation of the hydroxyl group at C-3;
✓ The A/B ring junction, which is trans in theonellasterol (**1**) and cis in CDCA;
✓ The unsaturation between C-8 and C-14 in theonellasterol (**1**);
✓ The lack of the carboxylic group at C-24 and the presence of an aliphatic side chain.

Of interest, in mammals, the LBD of FXR has a curved shape suitable for binding the bent steroidal core of 5β-bile acids (Figure 5), and the identification of a flat-shape steroidal molecule as a highly selective FXR antagonist represented a cornerstone in the decodification of the mechanism of FXR modulation.

Docking studies, elucidating the binding mode of theonellasterol (**1**) in FXR LBD, confirmed that, even if the A/B ring trans junction causes a different spatial arrangement, the marine sterol competes with 6-ECDCA (**42**), establishing several hydrophobic interactions within the LDB [18].

In addition, theonellasterol (**1**) attenuates liver injury caused by bile duct ligation, according to the measurement of serum alanine aminotransferase levels and the extent of liver necrosis at histopathology [18]. Analysis of genes involved in bile acid uptake and excretion by hepatocytes in this model revealed that theonellasterol (**1**) increases liver expression of MRP4, which, in contrast, is negatively regulated by FXR agonists. In summary, these studies demonstrate that FXR antagonism in vivo is feasible and results in positive modulation of liver MRP4 in rodent models of cholestasis [18]. This highlights the potential of marine organisms as a source of novel lead compounds for the treatment of human diseases.

Further pharmacological investigation of the secondary metabolites from *Theonella swinhoei*, collected at the Solomon Islands, allowed the identification of several 4-exo-methylene sterols as potent agonists of PXR and modulators of FXR [19]. In 2011, a library of polyhydroxysterols (theonellasterols B-H (**6**–**10**, **13**, **14**) and conicasterols B-D (**28**–**30**), Figure 2 and Figure 3) was isolated. Among these, theonellasterol G (**13**) increased the FXR target OSTα and simultaneously PXR target genes SULT2A1 and MDR1, resulting in the first example of FXR modulator and PXR agonist and, thus, a potential lead in the treatment of inflammatory bowel disease [19].

Docking calculations showed that, in addition to several hydrophobic interactions in the LDB of FXR, the β-orientation of the hydroxyl group at C-11 of theonellasterol G (**13**) is essential for the antagonistic activity [19]. With regard to the PXR agonistic activity, particularly crucial for the activation is the interaction between the 15α-OH group and Ser247 and the presence of the ethyl group at position C-24, engaging key interactions in the LDB [19]. This study disclosed, for the first time, marine steroids as dual modulators of PXR and FXR both involved in intestinal inflammation, paving the way towards the potential utility in the treatment of inflammatory bowel diseases.

Pursuing the systematic study on the chemical diversity of secondary metabolites from *Theonella swinhoei*, Sepe et al. isolated conicasterol E (**31**), a 7α,15β-dihydroxyconicasterol analogue. The pharmacological characterization of this sterol disclosed its activity as dual FXR/PXR modulator, able to induce gene expression of bile acids detoxification, such as BSEP and OSTα, without inducing SHP [37]. For the structural characterization of conicasterol F (**32**) and theonellasterol I (**15**), two other examples of dual FXR/PXR ligands, the traditional NMR analysis was not enough to uniquely establish their stereochemistry that has required the application of combined ROE–distance analysis and DFT calculations of the NMR chemical shifts [38].

By applying a chemoproteomic approach, in 2015, Margarucci and coauthors demonstrated that theonellasterone (**23**), 2-oxo-4-methylene-24-ethyl steroid, together with its antagonistic activity on FXR, is able to interact with peroxiredoxin-1 and to reduce enzyme cysteine overoxidation induced by H_2_O_2_ in both in vitro and in vivo living cells [39].

### 2.3. Unconventional Sterols as NRs Ligands

**Figure 6:**
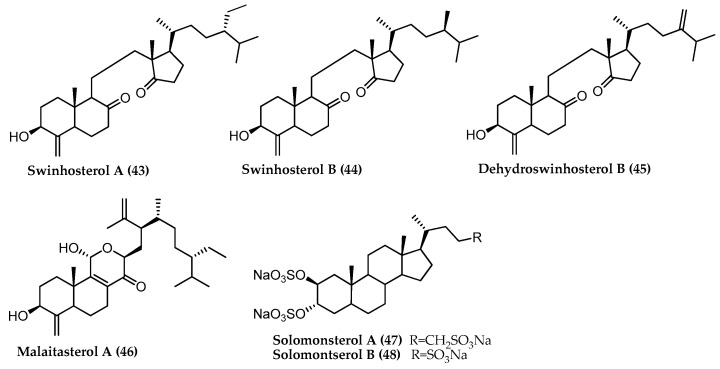
Swinhosterols, malaitasterol A, and solomonsterols from *Theonella*.

Steroids isolated from sponge are often characterized by the presence of unusual structural chemical features, such as additional oxygenation on the tetracyclic nucleus and on the side chain, sulfate esterification, alkylation or truncation of the side chain, unsaturation in the ring D, or secostructures with cleavage in the rings of the tetracyclic core [40]. This is the case of swinhosterols (**43**–**45**), unconventional steroids with the 4-exo-methylene and the 8–14 seco-8,14-dione functions (Figure 6). The structural modification of the basic carbon skeleton, with the cleavage of the six membered ring C, arises from the oxidation of the double bond between C-8 and C-14 [41].

In 2012, De Marino et al. reported the identification of conicasterols G-K (**33**–**37**) and theonellasterol J (**16**) (Figure 2 and Figure 3) together with some already known polyhydroxylated steroids. Among all isolated molecules showing dual PXR/FXR behavior, swinhosterol B (**44**) was selected as a potent PXR agonist/FXR antagonist. The ability of this marine sterol to induce the expression of target genes for PXR and FXR and to counter-regulate the induction of proinflammatory cytokines in a PXR-dependent manner was demonstrated [42].

Swinhosterols A (**43**) and B (**44**), together with the already reported theonellasterol (**1**) and conicasterol (**2**), showed also antagonistic activity towards ERRβ (estrogen-related receptor), another member of the nuclear receptor family, inhibiting the expression of the canonical target gene NKCC1 induced by genistein, similarly to diethylstilbestrol, a well-known ERR antagonist. Docking studies on swinhosterols within ERRs-LBD furnished the structural requirements for the interaction with the target [43].

Malaitasterol A (**46**), a potent PXR agonist isolated from a Solomon collection of *Theonella swinhoei* [44], presents a profound rearrangement in its steroidal core (Figure 6). Even if the 4-methylene group is already present, malaitasterol A (**46**) is characterized by the unprecedented 11,12–13,14-bis-secosteroid structure deduced by the analysis of spectroscopic data and arising from theonellasterol-like skeleton through the breaking of bonds in the C and D rings of the steroidal nucleus. The configuration at C-15 was established by DFT ^13^C chemical shift calculations.

Sulfated sterols, often isolated from marine sponge, are characterized by 2β,3α,6α-tri-O-sulfate groups and different patterns of substitution in the side chain. Festa et al. [11] isolated solomonsterols A (**47**) and B (**48**) from the butanol extract of a specimens of *Theonella swinhoei*, as the first example of truncated C-24 and C-23 side chains sulfated sterols of marine origin (Figure 6). These molecules, characterized by the presence of three sulfate groups (2 secondary and 1 primary) and a truncated C-24 or C-23 side chain, were demonstrated to be PXR agonists with a potency even higher than rifaximin, and therefore potential leads for the treatment of human disorders characterized by dysregulation of innate immunity [11]. Docking calculation showed that PXR allows accommodation of solomonsterols in its LBD, establishing several favorable hydrophobic interactions, hydrogen bonds between the C2-O-sulfate group and Cys284 and the sulfate on the side chain with Lys210 and electrostatic interactions with Ser247 (2-O-sulfate) and His407 (3-O-sulfate). All the above interactions contribute to the binding of the steroidal nucleus in the pocket of the nuclear receptor [11].

### 2.4. Sterols with Potential Anticancer Activity

The 4-exo-methylene sterols from *Theonella* have also attracted considerable attention for their cytotoxic activity.

The chemical analysis of *Theonella swinhoei* collected in the Philippines allowed the identification of the novel 7α-hydroxytheonellasterol (**4**), which showed, in vitro, an IC_50_ value (29.5 μM) higher than that of theonellasterol (IC_50_ > 100 μM), probably due to the presence of the additional 7α-OH group (Figure 2) [45].

In 2012, theonellasterol K (**17**), acetyltheonellasterol (**3**), and acetyldehydroconicasterol (**26**) were isolated from the specimens *Theonella swinhoei* collected from coral reefs off the coast of Pingtung in Taiwan (Figure 2 and Figure 3) [46]. Among these, theonellasterol K (**16**) exhibits cytotoxic activity against human colon adenocarcinoma (HCT-116), myelogenous leukemia (K562), and acute lymphoblastic leukemia (Molt 4) cancer cells.

**Figure 7:**
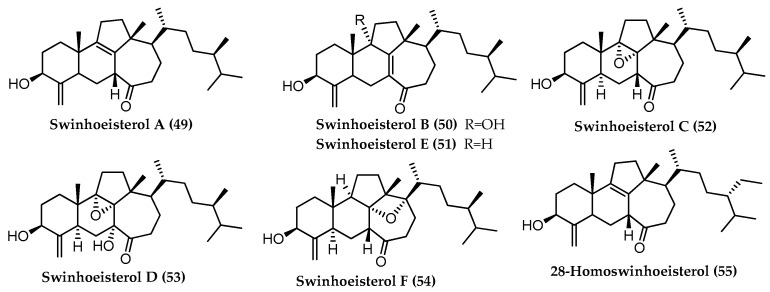
Swinhoeisterols from *Theonella swinhoei*.

Conicasterol L (**38**), dehydroswinhosterol B (**45**), and 7,15-oxoconicasterol (**39**) are three new compounds identified from *Theonella swinhoei* (Figure 2 and Figure 3); 7,15-oxoconicasterol (**39**) was tested on three human cancer cell lines and exhibited proapoptotic activity inducing accumulation of reactive oxygen species (ROS) within breast cancer cells [16].

In 2021, Lai et al. reported the isolation of theonellasterol L (**11**), together with three known 4-methylene sterols, two nucleosides, and one macrolide (Figure 2). The comparison of the cytotoxic activities of 4-methylene sterols reported in this paper with the previous reported ones showed that only derivatives highly functionalized and especially with oxygenated functions at position C-14 or C-15 are endowed with cytotoxic activity [47].

In 2020, Lee et al., in addition to revisiting the structure of 7α-hydroxytheonellasterol (**4**) identified for the first time by Faulkner in 2000, reported eight novel 4-exo-methylene sterols [48]. For 8α-hydroxy-14,15-β-epoxy-theonellasterol (**21**) (Figure 2), the β-orientation of the 14,15-epoxide was assigned by NOESY experiment and supported by GIAO NMR chemical shift calculations. The new isolated theonellasterol analogues did not show activity in anti-inflammatory assays.

Swinhoeisterols A (**49**) and B (**50**) from *Theonella swinhoei* collected off the cost of Xisha Island featured the unprecedented 6/6/5/7 ring system, expanding the family of sterols with rearranged carbon skeletons (Figure 7) [12]. As a consequence of an inverse virtual screening campaign, the biological activity of sterols from *Theonella* spp. was also expanded, demonstrating swinhoeisterols A (**49**) and B (**50**) as a new chemotype of (h)p300 inhibitors, a molecular target involved in several pathologies, mainly cancers.

Encouraged by the results obtained by swinhoeisterol A (**49**) (IC_50_ 3.3 μM vs. (h)p300), Zhan and collaborators reanalyzed the Xisha sponge *Theonella swinhoei*, [13] isolating four new swinhoeisterols, C–F (**51**–**54**) (Figure 7), with swinhoeisterol C (**52**) showing an inhibitory effect (IC_50_ 8.8 μM) towards (h)p300 like that of swinhoeisterol A (**49**). The biological results allowed delineation of a structure–activity relationship (SAR), suggesting the double bond or the epoxide function at C-8/C-9 to be essential for the activity towards (h)p300. On the contrary, the presence of an additional hydroxyl group at C-7 or a Δ^7^ double bond, as in swinhoeisterols D (**53**) and E (**51**), leads to the loss of activity.

## 3. Total Synthesis and Structural Modifications of *Theonella*-Inspired Sterols

### 3.1. Total Synthesis of Solomonsterols and Their Analogues

One of the main drawbacks of bioactive natural compounds is often the scarcity of isolated substances, hampering future developments. Unfortunately, even if marine natural products possess interesting and specific pharmacological activities, they often are obtained in insufficient amounts for preclinical and clinical testing.

The process to sample rare natural compounds, harvested from their natural source, can be laborious and in some cases, the total synthesis offers an alternative access.

This is the case for solomonsterols A (**47**) and B (**48**) (Figure 6), the first examples of marine sterols as PXR agonists [11]. Total synthesis of solomonsterols A (**47**) and B (**48**) was accomplished, furnishing the two natural compounds in large amounts for deeper pharmacological investigation and opening the way towards the development of a small library of derivatives. Structure–activity relationship studies facilitated information on the interaction between these leads and PXR and on their binding mode at atomic level [49].

**Scheme 1:**
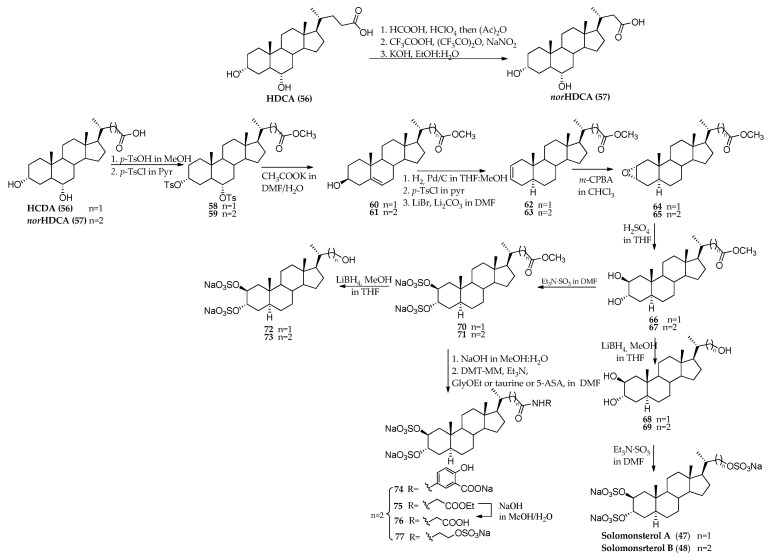
Synthetic route for preparation of solomonsterols A (**47**) and B (**48**), and analogues **72**–**74**, **76**, and **77**.

Starting from the commercially available hyodeoxycholic acid (HDCA, **56**), Sepe et al. [50] set up a robust synthetic route to prepare solomonsterol A (**47**) in large amounts. The same synthetic protocol starting from 24-*nor*-HDCA (**57**), in turn obtained by one-carbon degradation at C-24 on HDCA (isolated yield of 60% over the three-step sequence, (Scheme 1) [20,50], allowed access to solomonsterol B (**48**).

As depicted in Scheme 1, the key steps of the synthetic protocol are the modification of the functionalities on A/B rings to afford the desired trans junction and the installation of the hydroxyl groups at C2-β and C3-α. The required A/B trans ring junction was obtained through tosylation and simultaneous inversion at C-3 and elimination at C-6 (intermediates **60** and **61**). The introduction of the two hydroxyl groups at C-2 and C-3 on ring A was achieved through the introduction of Δ^2^ (intermediates **62** and **63**), epoxidation of the double bond (intermediates **64** and **65**), and subsequent epoxide opening providing the desired 2β,3α-diols (intermediates **66** and **67**). Finally, reduction at the methyl ester on the side chain and exhaustive sulfation of the alcohol functionalities afforded the desired molecules.

These synthetic routes were completed in 10 steps (31% yield) for solomonsterol A (**47**) and in a total of 13 steps (10% yield) for solomonsterol B (**48**), affording enough amounts for further pharmacological evaluation.

Tested in in vivo animal models of colitis, synthetic solomonsterol A (**47**) modulated the expression of proinflammatory cytokines TGFβ and IL10 by an NF-kB-dependent mechanism, and these findings make this compound a promising lead in the treatment of inflammatory bowel diseases (IBDs) [20].

In addition, solomonsterol A (**47**) was proven to be effective in attenuating systemic inflammation and immune dysfunction in a mouse model of rheumatoid arthritis [49].

However, the use of solomonsterol A (**47**) could cause severe systemic effects due to PXR activation in the liver. To overcome this limitation in clinical settings, a small library of derivatives was designed and prepared. Starting from the intermediates **66** and **67** (Scheme 1), the sulfation of C-2/C-3 diols followed by reduction or hydrolysis and coupling afforded the C-24 or C-23 alcohol derivatives (**72** and **73**) or the conjugate derivatives of solomonsterol A (**74**, **76**, and **77**) with 5-aminosalicylic acid, glycine, or taurine [51].

**Scheme 2:**
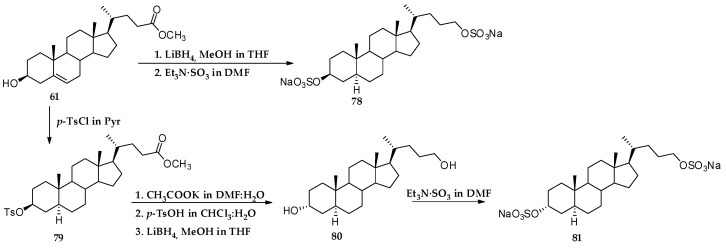
Synthetic route for preparation of compounds **78** and **81**.

**Scheme 3:**
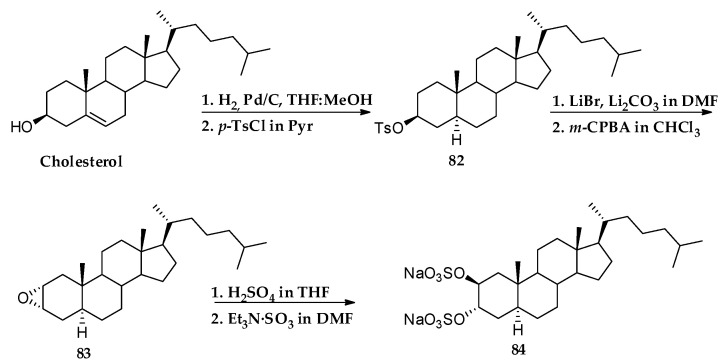
Synthetic route for preparation of compound **84**.

Similar modifications were made on intermediate **61** to speculate on the pharmacophoric role played by the functionalities on ring A in compounds **78** and **81** (Scheme 2), featuring the lack of the sulfate group at C-2 and bearing a 3β- or 3α-sulfate function, respectively. Starting from cholesterol, the same synthetic route used for the total synthesis of solomonsterols afforded cholestane disulfate (**84**) (Scheme 3) characterized by a hydrophobic side chain [51].

Cholestane disulfate **84** (Scheme 3), a simplified analogue of solomonsterol A (**47**), resulted to be the most promising compound coming from this medicinal chemistry campaign. This compound resulted to be a potent PXR agonist, able to increase the expression of the target gene CYP3A4 in HepG2 cells, similarly to the parent compound solomonsterol A (**47**). Further in vitro pharmacological evaluation demonstrated that compound **84** was able to modulate the immune response triggered by bacterial endotoxin in human macrophages and to reduce hepatic stellate cell transdifferentiation, affecting the basal expression of α-smooth muscle actin (αSMA) [51]. The above effects stated cholestane disulfate **84** as a new lead in the treatment of IBD [51] and liver fibrosis disease.

**Figure 8:**
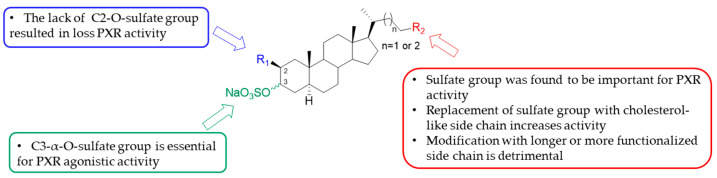
SAR of solomonsterol series [20,50,51].

The synthesis of these compounds allowed definition of an SAR (Figure 8). In particular, the length of the side chain bearing the sulfate group had no influence on the binding with PXR, whereas the alcohol derivatives in the side chain lost the ability (decreased the activity) to induce the expression of PXR target genes as well as the absence or inversion of the sulfate group at C-2 in ring A of the steroidal nucleus.

### 3.2. Structural Modifications of 4-Exo-Methylene Sterols

#### 3.2.1. Theonellasterol Series

**Figure 9:**
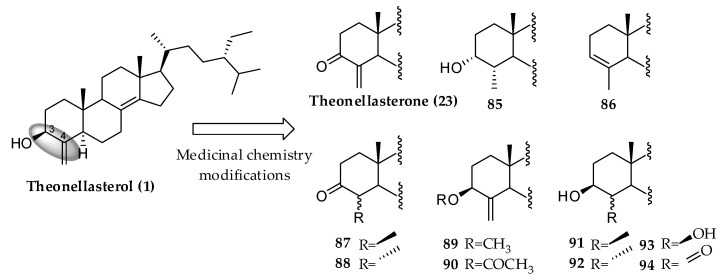
Modification on ring A of theonallasterol [52].

Chemical modifications on theonellasterol (**1**), a selective FXR antagonist [18], afforded a series of semisynthetic derivatives (Figure 9) [52]. In particular, the authors investigated the effect of chemical modifications on ring A of steroidal nucleus, regarding, particularly, the exo-methylene group at C-4 and the hydroxy group at C-3. The Δ^8,14^ bond was proven to be poor responsive to chemical modifications, therefore all derivatives maintained the above functionality.

**Figure 10:**
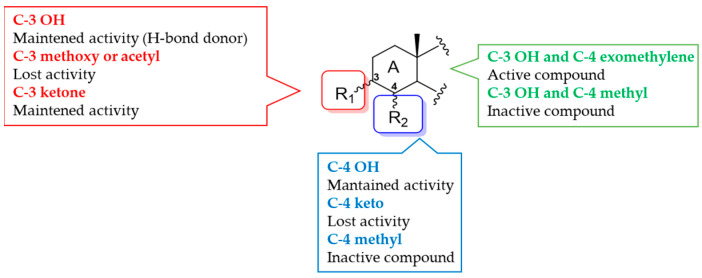
SAR of theonellasterol series [52].

This medicinal chemistry campaign allowed identification of compounds **87**, **88**, and **91** as the most promising leads and obtaining of fundamental information, also at atomic level using molecular docking studies, on the requirements necessary to maintain or lose activity towards FXR (Figure 10).

#### 3.2.2. Conicasterol Series

**Figure 11:**
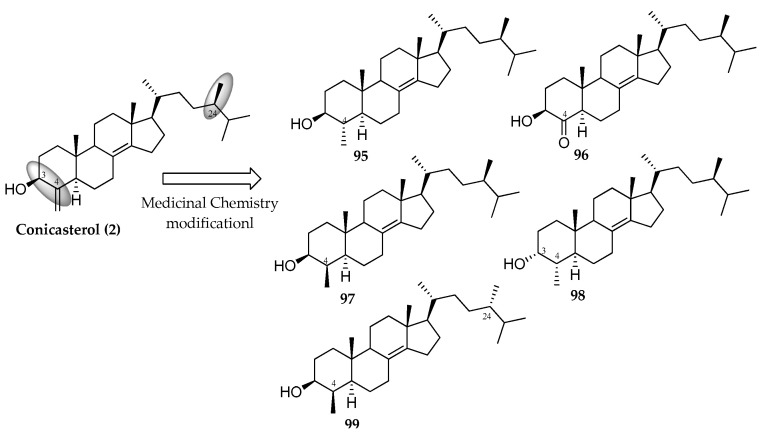
Semisynthetic conicasterol analogues [21].

Starting from conicasterol (**2**), with a significant PXR activating effect in HepG2 transfected cells [19,42], some modification on ring A and on the side chain afforded several 24-methyl semisynthetic derivatives (Figure 11) [21]. The combination of these chemical modifications, biological evaluation, and docking studies provided the molecular bases of ligand/PXR interaction, useful to delineate a preliminary structure–activity relationship.

In particular, the first series of modifications was made on the exo-methylene function at C-4, from the perspective of a total synthesis of more simplified and accessible PXR modulators inspired by conicasterol scaffold (Figure 11). This function was reduced affording the 4-α-Me derivative **95** or subjected to ozonolysis giving compound **96**. Oxidation of the hydroxyl group at C-3 followed by reduction of the C-4 exo-methylene functionality by catalytic hydrogenation and the ketone group by NaBH_4_ allowed access to compounds **97** and **98**, differing in the relative configuration of the substituents at C-3 and C-4. Finally, starting from dehydroconicasterol (**25**), the reduction of the double bond in the side chain afforded compound **99**, useful in exploring the importance of the configuration of the methyl group at C-24.

To delineate a SAR in PXR modulation, the above conicasterol semisynthetic derivatives, together with other natural sterols such as preconicasterol (**27**), 24-dehydroconicasterol D (**24**), and 25-dehydrotheonellasterol (**5**) (Figure 2 and Figure 3), were evaluated for their activity towards PXR. As a general trend, the substitution of 4-exo-methylene functionality with a methyl group (compounds **95**, **97**, and **99**) or the introduction of a keto group at C-4, as in **96**, causes a loss of activity towards PXR, except for compound **98**, featuring both substituents on ring A in α-configuration and retaining PXR agonistic activity. In addition, modifications on the side chain impacted on PXR activity, with a negative effect when the 24-ethyl or 24-exo-methylene groups were present, such as in 25-dehydrotheonellasterol (**5**) or 24-dehydroconicasterol D (**24**), respectively, while preconicasterol (**27**), bearing a cholestane-like side chain, maintained PXR agonistic activity.

### 3.3. Total Synthesis of Swinhoeisterol A (***49***) and Its Analogue (***105***)

**Scheme 4:**
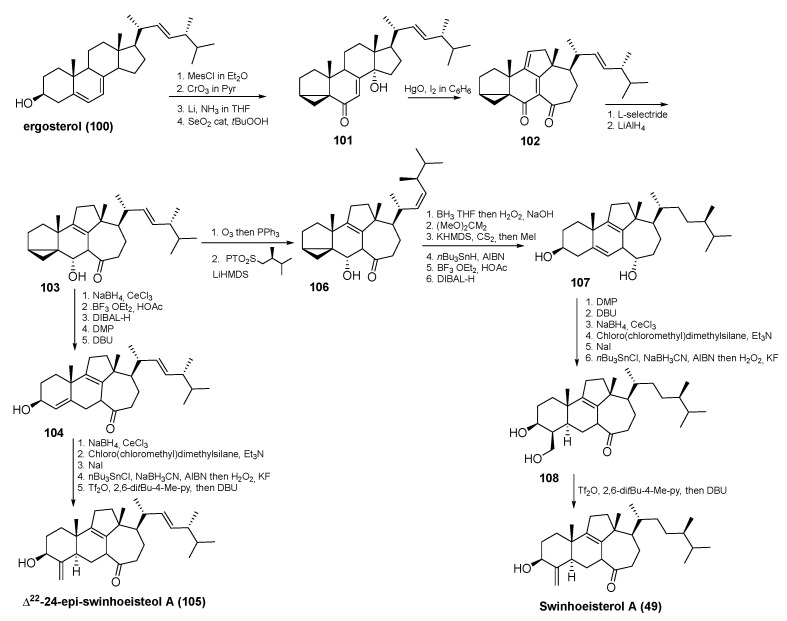
Synthetic route for preparation of swinhoeisterol A (**49**) and its semisynthetic analogue **105**.

In 2019, Duecker et al. reported, for the first time, the synthesis of swinhoeisterol A (**49**) from ergosterol by exploiting a radical framework reconstruction [53]. In addition, in 2020, the same authors described in detail the synthetic efforts (Scheme 4) towards the successful route of this unusual sterol and its analogue, Δ^22^-24-epi-swinhoeisterol A (**105**) [54]. As reported in Scheme 4, the key steps are the conversion of ergostane skeleton into 13(14→8)di*abeo* framework using a radical rearrangement of 14-hydroxy intermediate **101**, the introduction of campestane-like side chain in derivative **106**, and the installation of the 4-exo-methylene moiety via 4-hydroxy-methyl derivative **108**, followed by elimination.

## 4. Conclusions

Marine organisms, and especially sponges, have proven to be invaluable sources of metabolites with unique structures and diverse bioactivities such as antiviral, antimicrobial, antifungal, anti-inflammatory, and cytotoxic activities.

Secondary metabolites from sponges are the result of millions of years of evolution and natural selection. These molecules are synthetized through enzymatic reactions tuned towards the improvement of their capability to recognize and bind macromolecules, to perturb their activity, and to modulate biological processes. Considering that human protein targets contain structural domains similar to the targets with which sponge secondary metabolites have coevolved [23], the chemical, structural, and pharmacological characterization of small molecule libraries from sponges represents a greater promise to provide new drugs to treat human diseases.

*Theonella* sponges are known to produce several biosynthetic classes of secondary metabolites, comprising polyketides, cyclic and linear peptides, depsipeptides, alkaloids, pigments, lipids, sesquiterpenoids, and sterols. The exceptional chemical diversity found in metabolites from *Theonella* spp. has been attributed to the symbiotic microorganisms that the sponges host.

Excellent reviews, previously published, report the large number of bioactive metabolites found from worldwide collections of *Theonella* spp., mainly dominated by isolation of cyclic peptides [2,14].

In this review, we focused our attention on the natural, semisynthetic, and novel nature-inspired sterols from *Theonella* spp., summarizing their isolation, modification, and synthesis and highlighting their potentiality as candidates for the discovery of new therapeutic strategies for the treatment of different disorders, from liver and metabolic diseases to cancer. The possibility to delineate and clarify a structure–activity relationship (SAR) of this class of molecules and to investigate the effect of chemical transformations on the biological activity opens the way to the design and the preparation of new optimized analogues, more synthetically accessible and endowed with enhanced potency and selectivity as new drug candidates.

The investigation of these molecules reaffirms the role of natural products as essential chemical probes in today’s research arsenal, to shed light on complex biological processes and biochemical pathways, and in the identification of new therapeutical approaches to human diseases.

## Data Availability

Not applicable.
